# Turkish version of the academic and athletic identity scale: cultural adaptation and validation

**DOI:** 10.3389/fpsyg.2026.1783911

**Published:** 2026-04-08

**Authors:** Erkan Gulgosteren, Elif Yildirim, Mustafa Can Koc, Laurentiu-Gabriel Talaghir, Bogdan Sorin Olaru, Daniel Madalin Coja

**Affiliations:** 1 Faculty of Sport Sciences, Mersin University, Mersin, Türkiye; 2 Department of Statistics and Quality Coordinator, Konya Technical University, Konya, Türkiye; 3 Faculty of Sports Sciences, Istanbul Gelisim University, Istanbul, Türkiye; 4 Faculty of Physical Education and Sport, Dunarea de Jos University of Galati, Galati, Romania

**Keywords:** academic and athletic identity, cultural adaptation, scale, Turkish version, validation

## Abstract

**Background:**

This study aimed to adapt and validate the Turkish version of the Academic and Athletic Identity Scale (AAIS-Tr) for use among Turkish students studying at the faculty of sports sciences.

**Methods:**

The sample of the study consists of students studying at the faculty of sports sciences of a university in Türkiye, selected through convenience sampling. The original AAIS was translated into Turkish, and its validity and reliability were assessed. Internal consistency was assessed using Cronbach's Alpha coefficient and confirmatory factor analysis was used to confirm the two-factor structure obtained in exploratory factor analysis. Finally, measurement invariance was tested according to the gender of the participants to examine whether the scale was equivalent across different groups.

**Results:**

The reliability analysis revealed a Cronbach Alpha coefficient of 0.95 for the overall AAIS-Tr scale, with sub-scales “Academic Identity” and “Athletic Identity” showing coefficients of 0.911 and 0.962, respectively. The CFA results indicated acceptable fit indices: *χ*^2^/df (2.069), GFI (0.908), IFI (0.970), TLI (0.960), CFI (0.970), RMSEA (0.091), and SRMR (0.042). Measurement invariance analysis confirmed that the scale’s item-factor structure, factor loadings, intercepts, and error variances were equivalent across male and female participants, with ∆CFI and ∆RMSEA values within acceptable limits.

**Conclusion:**

The AAIS-Tr scale was found to be a valid and reliable tool for assessing academic and athletic identity among Turkish university students.

## 1 Introduction

The use of scales developed in one cultural context requires careful adaptation before being applied to another population. Cultural, linguistic, and educational differences may influence how individuals interpret scale items and respond to them, which can threaten the validity and reliability of the measurements. Therefore, cross-cultural adaptation and validation are essential to ensure conceptual, semantic, and measurement equivalence between the original and the adapted versions of a scale (Byrne, 2016; Herdman et al., 1998). Moreover, adapting existing instruments to new cultural contexts allows researchers to conduct comparable studies across different populations while ensuring that the constructs are accurately measured within the target culture. The concept of identity, that is a dynamic process shaped by the individual’s past experiences, current roles, and future related expectations, denotes a multidimensional approach that is regarding how the individual defines themselves, how she constructs themselves based on the relationships with her social environment, and how this process evolves over time (Geijsel and Meijers, 2005).

In the literature of social psychology, identity is defined as a fundamental structure that is constructed using interactional relationships of the individual with her social environment; and this structure is directly related to social roles that determine self-perception (Burke and Stets, 2009; Serpe and Stryker, 2011; Stets and Serpe, 2013).

The concept of ‘identity’ developed within this theoretical framework has become more distinct through diversified sub-identities such as academic, professional, and athletic in various contexts. Types of identity constructed in specific contexts by individuals have gradually gained more importance.

In this context, individuals’ academic identities are interrelated with how they define themselves during their higher education experience and how these definitions affect their processes of learning, achievement, and professional development (Henkel, 2000).

On the other hand, as a reflection of the emotional and cognitive connections of the individual with sports, athletic identity may be a determining factor in the identity structures of individuals who are intensely or professionally engaged in sports (Brewer et al., 1993).

Specifically, sports science students must develop these two dimensions of identity simultaneously and are confronted with high-performance expectations in both academic and athletic areas.

In the literature, the fact that these two identity dimensions are interwoven structures that are mostly interconnected rather than distinct and unrelated, has gained prominence (Settles, 2004; Lally, 2007). Thus, the ability to assess levels of academic and athletic identities of individuals separately is of great importance in understanding the developmental processes of students from sports disciplines because both academic and athletic fields place high-performance demands on them that contribute to their academic and athletic identities influencing each other. As a result, these students experience difficulty in balancing their two identities. That identity has a multidimensional structure, and individuals construct different identity components according to their various life roles. This has highlighted the importance of analyzing identity construction processes, especially in young adulthood.

As a result of this need, Yukhymenko-Lescroart (2014) developed the Academic and Athletic Identity Scale, which enables separate measurements of individuals’ academic and athletic identities. However, a commonly accepted principle in psychometrics is that each measurement tool needs to be adapted to different cultural contexts (Hambleton et al., 2005).

Limited research exists on the Turkish adaptation of the Academic and Athletic Identity Scale in the Turkish context. This gap both confines academic studies to a narrower scope and signals the insufficiency of quality tools for application.

Thus, a culturally adapted scale for university students in Türkiye will deepen their understanding of the field of study and fill a prominent gap in the field of application.

In this regard, adapting the Academic and Athletic Identity Scale with consideration of Turkish culture and language will lay the groundwork for studies on the processes of Turkish university students’ academic and athletic identity development. It is also expected to contribute to deepening academic studies and filling a significant gap in the field of application.

## 2 Materials and methods

### 2.1 Participants

This cross-sectional study, in accordance with the Helsinki Declaration, was conducted with students from Istanbul Gelişim University’s Department of Recreation in Sport Sciences as participants. Although the literature suggests different approaches to sample sizes for studies with factor analysis, the most valued method is to include five to ten times as many participants as the number of scale items (Osborne and Costello, 2019).

Accordingly, the sample size was 110 for this Turkish scale adaptation study, comprising 11 items. This estimation was based on the commonly recommended subject-to-item ratio of at least 5–10 participants per item for factor analysis studies. Therefore, the minimum required sample size ranged between 55 and 110 participants, and the final sample of 130 participants exceeded this recommended threshold, indicating that the sample size was sufficient for the planned factor analyses.

In order to tolerate missing and incomplete data, all students in the population were sent the form and actual 138 participants were chosen through convenience sampling, one of the non-probability sampling methods. Although convenience sampling is a non-probability method, it is frequently used in scale adaptation and validation studies where the primary aim is to evaluate the psychometric properties of a measurement instrument rather than to estimate population parameters. In such studies, the focus is on testing the reliability, validity, and internal structure of the scale within a relevant target population. Therefore, recruiting participants from the population of recreation students was considered appropriate for the validation of the AAIS-Tr.

The participants were included based on the following criteria: completing the form in full, being of Turkish ethnicity, and completing the consent form. The students who did not meet the inclusion criteria were excluded from the study. Out of 138 students that completed the form, 4 of them for not being of Turkish ethnicity, 3 of them for not completing and/or missing some parts of the form, and 1 for not filling out consent form were excluded, and the study was conducted with 130 participants in total.

### 2.2 Instruments and procedure

Adapting this scale to Turkish may affect students’ interpretation and response to the scale items due to cultural and educational differences. Based on this, the process has begun. The original AAIS that was developed by Yukhymenko-Lescroart (2014) consists of 11 items that are grouped into two subscales: academic identity (AI) (items 1–5) and athletic/sports identity (SI) (items 6–11). In this 7-point Likert scale, each item is rated from “It does not have a central role in understanding who I am (1)” to “It is a central component for me to understand who I am (7)” and the score is calculated by taking the average of relevant items for each subscale. The validity and the reliability studies of the original scale were conducted with undergraduate student athletes’ participants. In the Turkish context, the AAIS-Tr scale was adapted as described below to determine recreation students’ academic and athletic identities. Firstly, the adaptation process started by translating the scale into Turkish after getting the consent form from the developer of the relevant scale. Translation and back-translation methods were applied for the language adaptation and three linguists that are experts in English linguistics translated the scale into Turkish. Secondly, Turkish forms were translated from the target language to the original by a linguist experienced in adapting scales, and consistency between the original English and Turkish forms was achieved. Finally, to check the language and content validity of the ultimate Turkish form that a different researcher created, six experts (three language experts and three sports sciences experts) separately assessed the language and content validity. If the item is suitable “4,” suitable but needs minor changes “3,” somewhat suitable and needs major changes “2,” and not suitable “1” were scored. Content Validity Index (CVI) values were calculated using the Davis method. According to this method, CVI values are calculated by comparing the number of the experts that scored “3” and “4” for the scale items to the total number of experts as a ratio, and 0.80 is accepted as a criterion (Davis, 1992). After analyses, item CVI (I-CVI) values of items 1, 5, 7, 8, and 10 were calculated as 0.83, and the values of the other items were calculated as 1.00. To assess the overall content validity of the scale, the scale CVI (S-CVI) value was calculated by dividing the total CVI values of each item by the total number of items. 0.91 was found as S-CVI. The overall item and scale validity were determined to be good, and the last version of the scale was formed.

### 2.3 Statistical analysis

**Figure 1:**
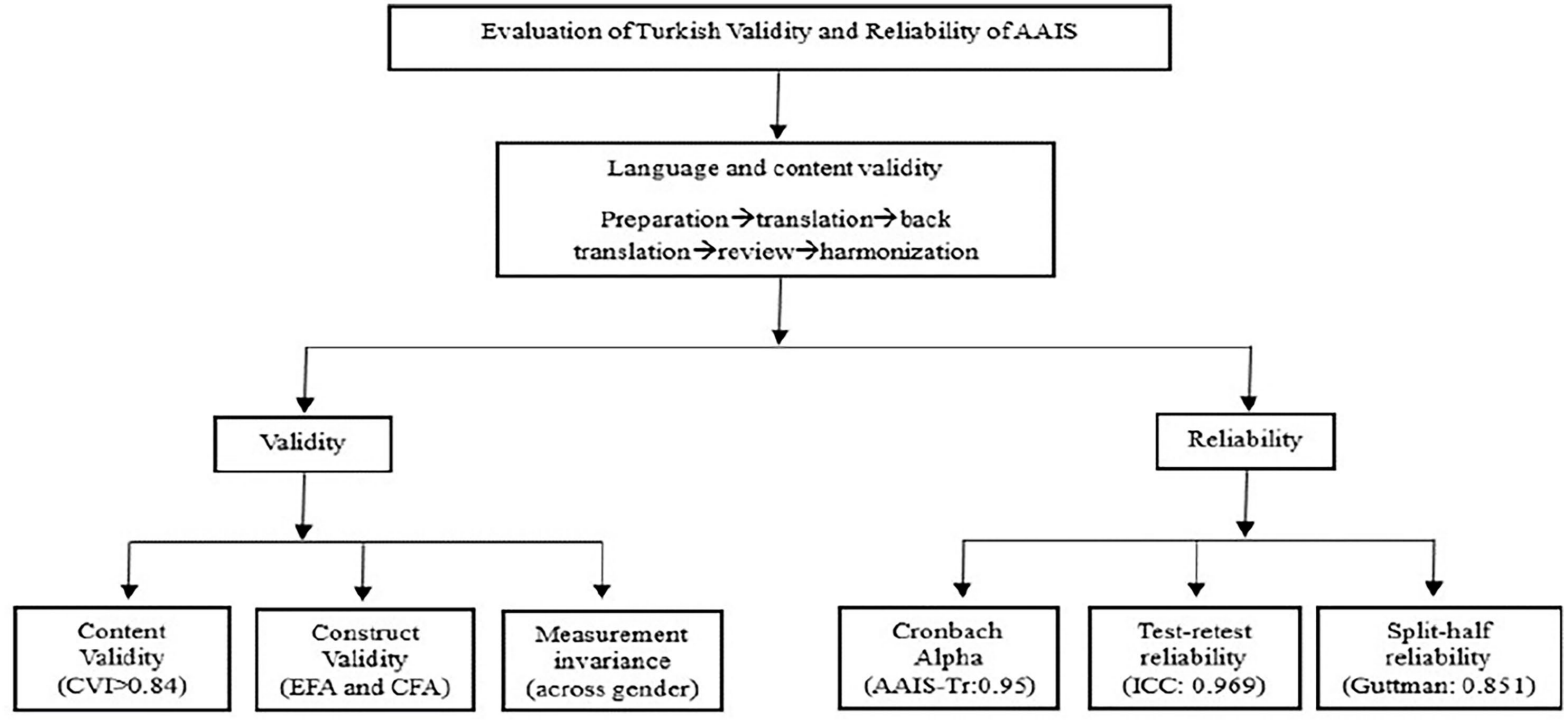
AAIS-Tr adaptation process.

The data was analyzed using SPSS 25.0 and version 4.0.5 of the R package. Firstly, expert opinions regarding the items were analyzed to assess the language and content validity of the Turkish version of the scale. The internal consistency of the scale was computed by using the Cronbach Alpha coefficient, which establishes a high level of reliability with scores of 0.70 and over (Davis, 1992). In addition, McDonald’s omega coefficients and corrected item–total correlations were calculated to provide a more comprehensive evaluation of internal consistency. Test–retest data was tested using Pearson correlation to determine the ability of the scale to provide consistent results between applications and their stability over time. To this aim, test–retest and internal consistency reliability analyses were used to analyze the scale’s reliability. The level of reliability was determined by calculating the reliability coefficient of the latest version of the scale. Before factor analysis, skewness and kurtosis coefficients were examined to assess missing data at the item level and distribution assumptions. The content validity was analyzed using exploratory factor analysis (EFA), and the suitability of the data for factor analysis was evaluated using the Kaiser–Meyer–Olkin (KMO) measure and Bartlett’s test of sphericity. Parallel analysis was performed to determine the number of factors. Since the factors were theoretically expected to correlate, principal axis factoring with promax rotation was used in EFA. Items with factor loadings below 0.40 and items with a difference of less than 0.10 between two-factor loadings were to be excluded (Tabachnick and Fidell, 2001). The confirmatory factor analysis (CFA) was applied to test the reliability of the structure that was found using EFA, and how well the data fitted the model was analyzed by using chi-square/degrees of freedom ratio (*χ*^2^/df), root mean square error of approximation (RMSEA), comparative fit index (CFI), standardized root mean square residual (SRMR), Tucker-Lewis index (TLI), goodness-of-fit index (GFI), and incremental fit index (IFI) (Simsek, 2007; Harrington, 2008; Lobiondo-Wood and Haber, 2010). Additionally, composite reliability (CR) and average variance extracted (AVE) values were calculated to assess convergent validity. The measurement invariance across gender was checked to investigate whether the scale provides the same measurement structure in different groups. The adaptation process of AAIS-Tr is summarized in Figure 1.

## 3 Result

According to the data gathered from 130 participants who met the study’s inclusion criteria, 39.2% of participants are female, and 60.8% are male. Gender was chosen as the demographic variable to test the measurement invariance in this Turkish adaptation study of the AAIS scale.

### 3.1 Exploratory factor analysis

According to EFA analysis results, which were conducted to identify whether the original scale and the adaptation of the scale for Turkish culture have the same structure, all the items have factor loading above 0.40, and they are not cross-loading items. Before factor extraction, missing data were examined and no missing values were found for any item. Furthermore, item means ranged from 4.09 to 4.59, and standard deviations ranged from 1.78 to 2.11. Since skewness values ​​ranged from −0.270 to 0.159 and kurtosis values ​​ranged from −1.481 to −1.075, it was determined that the item distributions satisfied the normality assumption. Accordingly, the general Kaiser-Meyer-Olkin (KMO) value of the Turkish version of the scale was found to be 0.91, and Bartlett’s test of sphericity was found to be statistically significant [*χ*^2^ (55) = 1461.45, *p* < 0.001]. These results showed that the correlation matrix was suitable for factor analysis. Parallel analysis suggested a two-factor solution. According to principal axis factoring results, there are two factors consistent with the original structure, and the variance explained by these two factors was determined to be 75.44% for the scale. Since the correlation between the subdimension is *r* = 0.68 (since *r* > 0.30), Promax rotation, an oblique rotation method, was applied to factor loadings to facilitate the interpretation of the factors (Taherdoost et al., 2014). It is presented in detail in Table 1. Both the original scale and the adapted version have two subdimensions. Items 1–5 are the AI subdimension, and items 6–11 are the SI subdimension (Table 1). Factor loadings for the AI subdimension ranged from 0.651 to 0.988, whereas factor loadings for the SI subdimension ranged from 0.790 to 0.948. Cross-loadings were generally low, supporting a clear two-factor structure. The descriptive statistics related to the scale and Cronbach alpha values of the subdimensions are provided in Table 2.

**Table 1:** Factor loadings obtained by promax rotation.

Item	Factor 1	Factor 2
Being a capable student		0.651
Being satisfied with my academic work		0.824
Doing well in school		0.988
Getting good grades		0.729
Having high GPA		0.798
Being a capable athlete	0.790	
Being a good athlete	0.948	
Being athlete	0.889	
Being proud to be an athlete.	0.897	
Being satisfied with my athletic achievements	0.862	
Doing well during sport competitions	0.914	
Eigenvalue coefficient	4.883	3.415
Explained variance	44.39	31.05
Explained variance (cumulative)	44.39	75.44

**Table 2:** Descriptive statistics and reliability values for the scale and its sub-dimension.

Scale/sub-dimension	Mean	S. D.	Min.	Max.	Cronbach Alpha	McDonald’s Omega
AI	4.166	1.580	5	35	0.911	0.913
SI	4.510	1.828	6	42	0.962	0.963
AAIS-Tr	4.354	1.573	11	77	0.950	0.966

### 3.2 Confirmatory factor analysis

**Figure 2:**
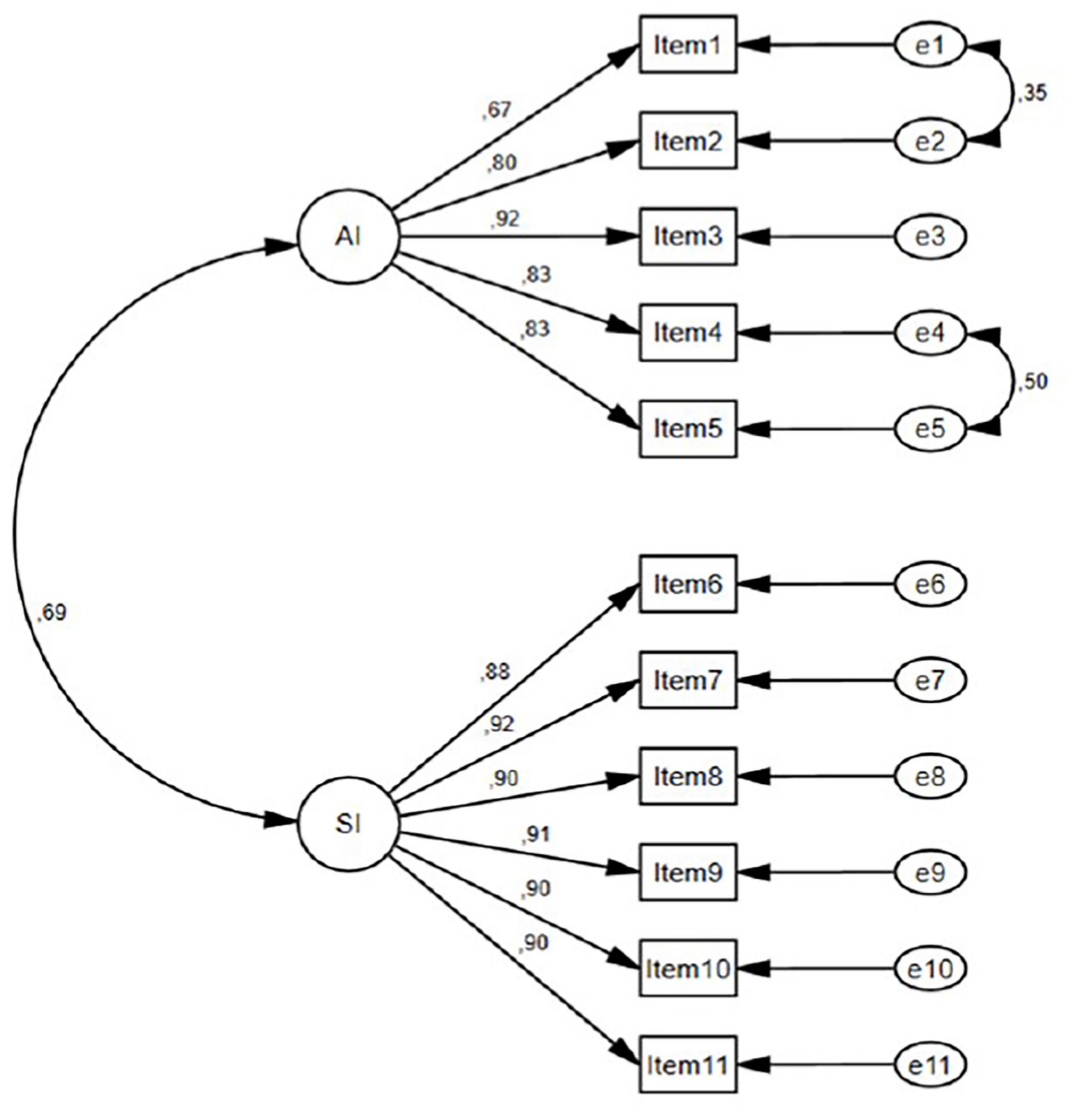
Path diagram of AAIS-Tr.

To adapt the AAIS scale for Turkish society and to confirm this two-dimensional structure obtained through EFA analysis, CFA was applied to 130 students, the Cronbach alpha value was found to be 0.95, and the KMO value was determined to be 0.91. The Cronbach alpha coefficients of AI and SI subdimensions are 0.911 and 0.962, respectively. In conclusion, the 11 items and two subdimensions of the AAIS scale were determined to be highly reliable for Turkish society. In addition, McDonald’s omega coefficients were calculated as 0.966 for the total scale, 0.913 for the AI subdimension, and 0.963 for the SI subdimension. Corrected item–total correlations ranged from 0.599 to 0.842, indicating that all items contributed adequately to the overall construct. Furthermore, as a result of the split-half method for the scale, the Cronbach alpha coefficient for the first half (6 items) was 0.912, and for the second half (5 items), 0.958. The correlation between split halves was determined to be 0.741. According to the split-half method, the reliability coefficients were calculated using Spearman-Brown (0.851 for equal lengths and 0.852 for unequal lengths) and Guttman split-half coefficient (0.851). All these coefficients are above 0.80, and it was concluded that the scale is highly reliable. Additionally, test–retest reliability was assessed with 40 participants who did not participate in the study. AAIS-Tr was applied with a three-week time interval to test temporal invariance. The intraclass correlation coefficient related to test–retest reliability found on 40 students who participated in both studies was calculated as 0.969, *p* < 0.001. Based on these results, the scale, consisting of 11 items, was found to be reliable. CFA fit indices were calculated to test the reliability of the two-factor structure obtained from EFA. According to the robust CFA results, the fit indices of AAIS-Tr were found to be scaled *χ*^2^ (43) = 74.38, *p* = 0.002, robust CFI = 0.962, robust TLI = 0.952, robust RMSEA = 0.098, and SRMR = 0.043. Standardized factor loadings ranged from 0.654 to 0.890 for the AI factor and from 0.876 to 0.917 for the SI factor. The correlation between the two latent factors was 0.709, indicating a substantial positive association between academic and athletic identity. In addition, convergent validity was supported by the CR and AVE values. CR values were 0.912 for AI and 0.963 for SI, and AVE values were 0.678 for AI and 0.811 for SI. According to the findings shown in Table 3 and Figure 2, fit indices are acceptable, and this shows that the adaptation of AAIS-Tr with its two-factor structure is acceptable and applicable to Turkish culture. However, the RMSEA value suggests that the model fit is not perfect and should be interpreted with some caution.

**Table 3:** AAIS-Tr model goodness of fit indices.

Fit index	AAIS-Tr	Acceptable fit	Perfect fit
$\chi^{2}$ /df	2.950	<5	<3
TLI	0.952	>0.90	>0.95
CFI	0.962	>0.90	>0.95
RMSEA	0.098	<0.08	<0.05
SRMR	0.043	<0.10	<0.05

Multi-group measurement invariance analyses of AAIS-Tr were conducted using maximum likelihood and gender variable based on the variance–covariance matrix. The measurement invariance was tested by the hierarchy of configural, metric, scalar, and strict invariance based on the suggestions of Byrne (2016). ∆CFI and ∆RMSEA values were considered to identify whether the measurement invariance was established between two hierarchical stages. In model comparisons, ∆CFI < 0.020 and ∆RMSEA < 0.030 thresholds for the configural and metric invariance, ∆CFI < 0.010 and ∆RMSEA < 0.015 thresholds for the metric and scalar invariance models as suggested by Rutkowski and Svetina (2014). Additionally, as a second criterion for all metrics, as suggested by Cheung and Rensvold (2002), ∆CFI and ∆RMSEA < 0.01 threshold across a hierarchical ranking of the models was considered for the strict invariance model and other models.

Configural invariance tests whether the scale has the same factor structure in male and female groups. According to Table 4, fit indices of the configural model were *χ*^2^/df = 3.27, CFI = 0.874, TLI = 0.839, RMSEA = 0.187, SRMR = 0.064. Although the RMSEA value is above the acceptable level, the configural model still provided an initial basis for subsequent invariance testing. Therefore, it is accepted that the configural invariance is achieved and is considered acceptable. Accordingly, it can be stated that the male and female groups have the same factor structure.

**Table 4:** Fit statistics for measurement invariance according to gender.

Model	$\chi^{2}$	df	$\chi^{2}/\mathrm{df}$	CFI	TLI	RMSEA	SRMR	∆CFI	∆RMSEA
Configural	281.612	86	3.274	0.874	0.839	0.187	0.064	—	—
Metric	287.813	95	3.030	0.876	0.857	0.177	0.062	0.002	−0.010
Scalar	289.593	104	2.785	0.881	0.874	0.166	0.062	0.005	−0.011
Strict	307.208	115	2.671	0.877	0.882	0.160	0.062	−0.004	−0.005

Metric invariance tests whether factor loadings are the same across groups, and it has critical importance as it evaluates whether the underlying structure of the scale functions the same way across groups. When the model results are compared to the configural model, it is observed that ∆CFI = 0.002 and ∆RMSEA = −0.010 values are below the threshold values that Rutkowski and Svetina (2014) suggest. Accordingly, it can be stated that metric invariance is achieved, and factor loading is equal across groups.

Scalar invariance assesses whether the items’ intercepts and factor loadings are equal across groups. This stage is crucially important to make meaningful comparisons between groups. The values obtained by comparing the metric model to the scalar model were below the threshold values (∆CFI = 0.005 and ∆RMSEA = −0.011). These findings show that scalar invariance is achieved, and the intercepts of the items are equal across groups.

Strict invariance examines whether factor loadings, intercepts, and error invariances across groups are equal. This stage is crucial as it shows that the scale is completely invariant. When the model results are compared to the scalar model, it is found that the obtained values are below threshold values (∆CFI = 0.004 and ∆RMSEA = −0.005). As a result, it can be put forward that the error variances do not change across gender groups.

Overall, according to the goodness of fit statistics obtained through the analysis using the multi-group CFA method, the changes in fit indices across increasingly constrained models were very small (∆CFI ≤ 0.005; ∆RMSEA ≤ 0.011), suggesting approximate measurement invariance across gender. Based on this, it can be concluded that item-factor structure and factor loadings, variances, covariances, and error variances are largely equivalent between male and female groups. Given the relatively weak absolute fit of the configural model, these invariances results should be interpreted cautiously.

## 4 Discussion

This study aims to adapt AAIS to Turkish culture and provide a valid and reliable tool to assess the awareness of academic and athletic identities of sports sciences students specifically in Türkiye. Firstly, it was translated into Turkish, and language and content validity were tested. Then, the last version of the scale is formed by considering the experts’ suggestions. The construct validity of AAIS-Tr was examined with EFA, and the findings suggest that AAIS-Tr also has a two-factor structure like the original scale. Factors are named AI and SI, like the original scale. The adequacy of the dataset for factor analysis was supported by an excellent KMO value (0.91) and a significant Bartlett’s test of sphericity. In addition, parallel analysis supported the retention of two factors, and the EFA findings showed that all items loaded strongly on their expected factors with low cross-loadings.

The findings of this study are consistent with the original scale developed by Yukhymenko-Lescroart (2014), which has a two-factor structure consisting of academic identity and athletic identity. Similar findings were obtained with the original study, revealing that academic and athletic identities represent closely related but conceptually distinct components of students’ self-concept. The preservation of this two-factor structure in the Turkish adaptation demonstrates that the theoretical framework underlying the original scale is also applicable within Türkiye’s cultural and educational context.

The scale’s reliability is tested using Cronbach alpha, test–retest, and split-half methods. In response to the reviewers’ recommendations, McDonald’s omega coefficients, corrected item–total correlations, CR, and AVE values were also calculated, and all findings supported the internal consistency and convergent validity of the scale. The two-factor structure obtained through EFA was examined using CFA, and it has been determined that the scale demonstrated generally acceptable model fit according to the fit index coefficients.

Multi-group CFA analysis was conducted to test the measurement invariance of the scale across male and female students. Configural invariance showed that the scale’s factor structure is the same across groups. Metric invariance demonstrated that factor loadings are equal across groups. Scalar invariance indicated that intercepts of the items are the same across groups. Moreover, strict invariance confirmed that error variances are equivalent between groups. Hence, it has been identified that the scale is comparable between male and female students, and the measurement invariance was ensured. However, because the absolute fit of the configural model was not ideal, the invariance findings are better interpreted as evidence of approximate rather than perfect invariance across gender.

The study found that AAIS-Tr is a valid and reliable tool for assessing the awareness of sports science students’ academic and athletic identities. This 7-point Likert-type scale has 11 items and two subdimensions: AI (items 1–5) and SI (items 6–11). The minimum and maximum scores obtained from the scale are between 11 and 77. High scores show that the students’ awareness of their academic and athletic identities is high.

Aydın and Aslan (2024) introduced the teacher identity scale into Turkish literature, filling a gap in the literature. However, there is still a gap in academic and athletic identity. This Turkish adaptation study aims to fill this gap. Many studies are expected to follow after this scale contributes to Turkish culture. Beyond the psychometric contribution of this scale, AAIS-Tr can also serve as a useful assessment tool for researchers and practitioners working with sports science students. Understanding the balance between students’ academic and athletic identities can help universities design more effective academic support systems and athlete development programs. Furthermore, the scale can help identify students who struggle to balance their dual roles as both student and athlete.

## 5 Conclusion

Although AAIS-Tr was found to be valid and reliable for Turkish society, it has some limitations. One important limitation of the study is that the sampling procedure relied on convenience sampling and was conducted within a single institution. Nevertheless, it has been found that AAIS-Tr is a valid and reliable tool for examining the awareness levels of Turkish sports science students about their academic and athletic identities. However, to generalize the results, the study should be conducted again with a larger sample, including university students from different parts of Türkiye. As this study focuses on university students, future research might examine the applicability of the AAIS-Tr to different populations, such as sports high school students and professional athletes. Another limitation is that, although the two-factor structure was supported, some model fit indices suggest that the model fit was not perfect. Therefore, future studies should re-examine the factor structure with larger and more diverse samples.

Despite these limitations, AAIS-Tr is a highly reliable and valid tool to measure the academic and athletic identities of Turkish sports science university students. The scale might be used as a practical tool because of its short length and strong psychometric properties. In future studies, AAIS-Tr might be used to find relationships with other variables, such as academic performance, athletic success and psychological well-being, and academic and athletic identities. Moreover, it might contribute to the literature regarding the comparisons of academic and athletic identities of Turkish students and other sports science students in other countries and the role of cultural factors in shaping identity construction.

In conclusion, AAIS-Tr provides a psychometrically sound tool for investigating the academic and athletic identities of Turkish sports science students and examining the underlying relationships. The usability of this scale may support future researchers investigating the interaction between academic participation, athletic commitment, and psychological well-being in student-athlete populations in Turkey. Furthermore, it may contribute to cross-cultural comparisons of identity structures among student-athletes in different educational and cultural contexts.

## Data Availability

The raw data supporting the conclusions of this article will be made available by the authors, without undue reservation.
